# Supplementary material for: Anticancer Activities of Novel Nicotinamide Phosphoribosyltransferase Inhibitors in Hematological Malignancies
Source: Molecules. 2023 Feb 16;28(4):1897. doi: 10.3390/molecules28041897 (PMC9967653; doi:10.3390/molecules28041897)
Supplement: Supplementary file 1 [file molecules-28-01897-s001.zip › molecules-2124986-supplementary.pdf]

Supplementary Materials

Table of Contents

Information on chemical synthesis of **JJ08** and its characrerization 3

Information on chemical synthesis of **FEI191**, **FEI199** and their characterizations 7

Geometric means of plasma concentrations of the NAMPT inhibitors... 14

Compounds concentrations used for the PK analysis.. 15

Syntheses of NAMPT inhibitors **JJ08, FEI191** and **FEI199.**

General Remarks

Unless specified, all commercially reagents were purchased from Sigma Aldrich, Fluka or Acros and used without further purification. For reactions requiring anhydrous conditions, dry solvents were bought (Fluka, Aldrich). All reactions were carried out under nitrogen or argon atmosphere in oven-dried glassware with magnetic-bar stirring. Analytical TLC (thin layer chromatography) was performed with silica gel 60 F254 aluminum plates (Merck) with visualization by UV light and charring with aqueous $KMnO_4$ solution, ethanolic ninhydrin solution or Pancaldi reagent. Flash column chromatography (FC) was performed with 230-400 mesh, MN Kieselgel 60M silica gel (Merck). ^{1}H and ^{13}C NMR spectra were recorded with Bruker AVIII-400 spectrometer at 400 MHz and 100 MHz respectively at 20°C (the piperidine of amide show split signals in ^{13}C NMR at 20°C). Chemical shifts are calibrated using residual solvents signals ($CDCl_3$: δH = 7.26, δC = 77.16; CD_3CN: δC =1.94, δC = 118.26, 1.32) and reported in ppm (abbreviations: s = apparent singlet, br. = broad, d = apparent doublet, t = apparent triplet, q = apparent quadruplet, m = mutiplet). HRMS spectra were recorder on Mass Spectral Facility of the Institute of Chemical Sciences and Engineering (ISIC), Swiss Federal Institute of Technology, Lausanne (EPFL) and are given in m/z.

Part A: Synthesis of (*E*)-*N*-(4-(1-(furan-2-carbonyl)piperidin-4-yl)butyl)-3-(pyridin-3-yl)acrylamide (**JJ-08**).

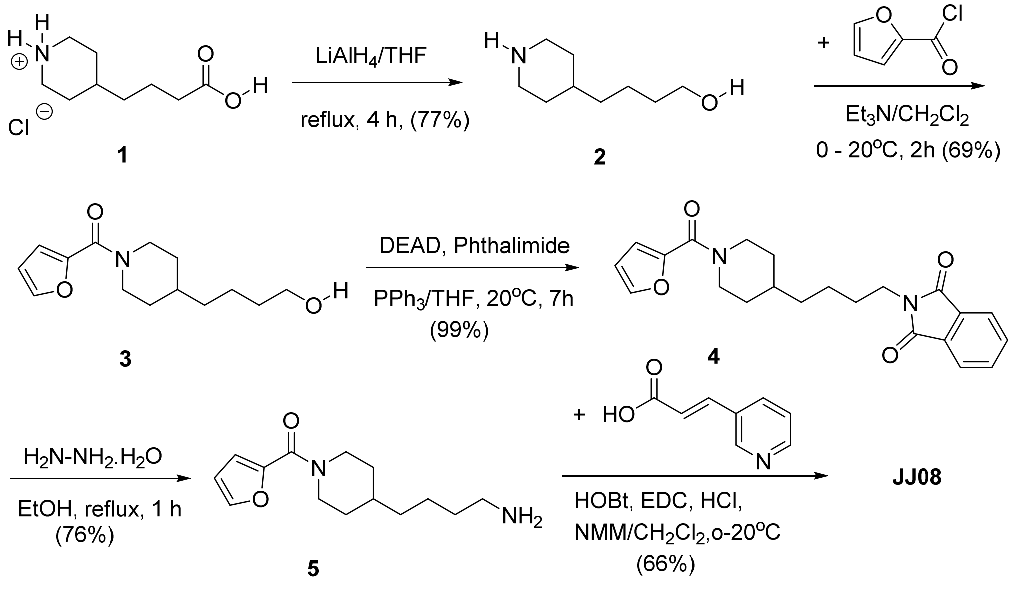

4-(Piperidin-4-yl)butanol (2). A suspension of 4-(piperidin-4-yl)butyric acid hydrochloride (1, MW: 206.7) (5.04 g, 0.024 mol) in dry THF (220 mL) was cooled in an ice bath and stirred during the portionwise addition of lithium aluminum hydride (MW: 37.95) (3.69 g, 0.097 mol). Stirring was continued at room temperature for 10 min and the reaction mixture was heated under reflux for 6 h. For TLC analysis, sample preparation is done with three drops of 40% aqueous KOH and EtOAc. After being soaked in a solution of $KMnO_4$, TLC plate ($MeOH/CH_2Cl_2$ 9:1), showed complete conversion of the starting material. The reaction mixture was cooled to 0°C, followed by the slow addition of aqueous 40% KOH over a period of 15 min under vigorous stirring. Stirring was continued at room temperature for 1 h. The suspension was filtered on a bed of Celite and the cake was washed with THF (100 mL) and MeOH (50 mL). After evaporation of the solvent *in vacuo*, the residue was purified by FC, first

eluting with EtOAc/Et_3N 98:2, and then with a gradient of EtOAc/Et_3N/MeOH from 78:2:20 to 18:2:80, UV detection at 254 nm. Collecting and evaporation of the fractions yielded **2** (MW: 157.239), yellow oil (2.69g, 71%).

^{1}H NMR (400 MHz, $CDCl_3$) δH: 3.64 (t, 2H, 3J (HC(1)-HC(2)) = 6.8 Hz, $H_2C(1)$, 3.14 (br. d, 2H, 2J(HC(2'a,6'a)-HC(2'e,6'e)) = 12.6 Hz, HC(2'a) & HC(6'a)), 3.03 (br. s, 1H, H-O), 2.62 (br. td, 2H, 2J(HC(2'a,6'a)-HC(2'e,6'e)) = 12.6 Hz, HC(2'e) & HC(6'e), 1.72 (br. d, 2H, 2J (HC(3'a,5'a)-HC(3'e,5'e)) =12.6 Hz, H-C(3'a) & HC(5'a)), 1.57 (m, 2H, 3J(HC(1)-H(2)) = 6.8 Hz, $H_2C(2)$), 1.48-1.10 (m, 8H, HC(2), $H_2C(3)$, H_2(C(4), HC(5), H-C(3'e) & HC(5'e)).

Furan-2-yl(4-(4-hydroxybutyl)piperidin-1-yl)methanone (3).

Under Ar atmosphere, TEA (1.5 mL, 1.07 mmol, 2 equiv.) and 2-furoyl chloride (0.50 mL, 5.06 mmol, 1 eq.) were subsequently added to a cooled (0°C) solution of **2** (800 mg, 5.09 mmol), in dry CH_2Cl_2 (14 mL) and the reaction mixture was stirred for 10 min. After stirring at room temperature for 1 h and 45 min. TLC (EtOAc/PE 8:2) analysis showed the reaction to be complete. Work-up began with dilution by using sat. aq. NH_4Cl until a clear solution was obtained, then the mixture was extracted with CH_2Cl_2 (30 mL, 3 times). The combined organic extracts were washed with brine (40 mL), dried over Na_2SO_4 and filtered. The solvent was evaporated and the residue was purified by FC (EtOAc/PE/TEA 70:28:2 as eluent) to give 880 mg (68.7%) of **3** (MW: 251.308), yellow oil used as such in the next preparation.

^{1}H NMR (400MHz, $CDCl_3$) δH: 7.49 (br. s, 1H, HC(5'), fur), 6.96 (d, 1H, 3J (HC(3')-HC(4')) = 3.6 Hz, H-C(3'), fur), 6.48 (dd, 1H, 3J(HC(3')-HC(4')) = 3.6 Hz , 3J(HC(4')-HC(5')) = 1.8 Hz, H-C(4'), fur), 4.53 (br. s, 2H, $H_2C(4''')$, hydroxybutyl), 3.68 (t, 2H, 3J(HC(2"a)-HC(3"a)) = 3J(HC(5"a)-HC(6"a)) = 5.7 Hz, HC(2"a) & HC(6"a), piper), 2.93 (m, 2H, HC(2"e) & HC(6"e), piper), 1.80 (m, 2H, $H_2C(3''')$, hydroxybutyl), 1.64-1.51 (br. m), 1.48-1.38 (br. m), 1.37-1.17 (br. m).

2-(4-(1-(Furan-2-carbonyl)piperidin-4-yl)butyl)isoindoline-1,3-dione (4).

Under Ar atmosphere, phthalimide (0.62 g, 0.42 mmol, 12 equiv.) and Ph_3P (1.10 g, 0.42 mmol, 1.2 equiv.) dissolved in dry THF (5 mL) were added to a solution of **3** (0.880 g, 3.5 mmol, 1 equiv.) in dry THF (8 mL). The reaction mixture was stirred at room temperature for 10 min and cooled to 0°C. After 10 min, DEAD (40%) (1.90 mL, 0.41mmol, 1.2 equiv.) was added dropwise to the reaction mixture under stirring and at 0°C. After the addition, the cooling bath was removed and the resulting solution was stirred at room temperature for 7 h. TLC (EtOAc/PE 8:2) analysis showed the reaction to be complete. The solvent was evaporated and the residue was purified by FC (PE/EtOAc 55:45) to give 1.33 g (99%) of **4** (MW: 380.424) as a yellow powder.

^{1}H NMR (400 MHz, $CDCl_3$) δH: 7.86 (dd, 2H, 3J(ortho) = 5.3 Hz, 3J(meta) = 2.9 Hz, HC(3) & HC(7), isoindol), 7.74 (dd, 2H, 3J(ortho) = 5.3 Hz, 3J(meta) = 2.9 Hz, HC(5) & HC(6), isoindol), 7.49 (br. d, 1H, 3J(H(4"')-HC(5"')) = 1.5 Hz, HC(5"'), fur), 6.95 (d, 1H, 3J(H(3"')-HC(4"')) = 3.5 Hz, HC(3"'), fur), 6.48 (dd, 1H, 3J(H(3"')-HC(4"')) = 3.5 Hz, 3J(H(4"')-HC(5"')) = 1.5 Hz, HC(4"'), fur), 4.52 (m, 2H, HC(2"a) & HC(6"a), piper), 3.71 (t, 2H, 3J(HC(3')-HC(4')) = 7.0 Hz, H2C(4'), but), 2.92 (m, 2H, HC(2"e) & HC(6"e), piper), 1.82-1.15 (br. m, 9H).

(4-(4-Aminobutyl)piperidin-1-yl)(furan-2-yl)methanone (5).

Under Ar atmosphere, **4** (1.33 g, 3.50 mmol) and hydrazine hydrate (0.43 mL, 0.85 mmol) were dissolved in dry ethanol (45 mL). The reaction mixture was stirred at room temperature for 10 min and heated under reflux (85°C). After 4 h, TLC (EtOAc/PE = 8:2) analysis showed the reaction to be complete. During the reaction a white precipitate was generated (byproduct). Work-up consisted of removal of solid by filtration, washing the filter-cake with additional EtOH, and evaporation of the filtrate. The resulting slurry was dissolved in CH_2Cl_2 and K_2CO_3 solution was added to get a clear solution. The aqueous layer was washed with CH_2Cl_2 (20 mL, 3 times) and the combined organic extracts were washed with brine (50 mL). After drying over sodium sulfate, filtration, washing with

CH_2Cl_2 and evaporation of the solvent, the crude was purified by FC (MeOH/EtOAc/TEA 80:18:2) to give 670 mg (76.4 %) of **5** (MW: 250.352) as orange oil used directly in the next step.

^{1}H NMR (400 MHz, $CDCl_3$) δH : 7.49 (br. d, 1H, 3J(HC(4''')-HC(5''')) = 1.7 Hz, HC(5'''), fur), 6.95 (d, 1H, 3J(HC(3''')-HC(4''')) = 3.3 Hz, HC(3'''), fur), 6.48 (dd, 1H, 3J(HC(3''')-HC(4''')) = 3.3 Hz, 3J(HC(4''')-HC(5''')) = 1.7 Hz, HC(4'''), fur), 4.63-4.41 (m, 2H, HC(2"a) & HC(6'a), piper), 3.15-2.88 (m, 2H, HC(2"e) & HC(6'e), piper), 2.73 (br. t, 2H, 3J(HC(3")-HC(4")) = 6.9 Hz, H_2C(4"), 4-aminobutyl), 1.85-1.16 (m, 9H).

(*E*)-N-(4-(1-(furan-2-carbonyl)piperidin-4-yl)butyl)-3-(pyridin-3-yl)acrylamide (JJ-08).

Under Ar atmosphere, HOBt hydrate (MW: 135.1; 135 mg, 1 equiv.), EDCI/HCl (MW: 191.7; 200 mg, 1.04 equiv.) and acrylic acid (MW: 72.06; 80 mg, 1.1 equiv.) were sequentially added to a cooled (0°C) and stirred solution of **5** (MW: 250.35; 251 mg, 1 mmol, 1 equiv.) in dry CH_2Cl_2 (15 mL). The reaction mixture was stirred for 3 min and *N*-methylmorpholine (MW: 101.149; 0.55 mL, 5 mmol) was added dropwise maintaining the temperature at 0°C. The resulting reaction mixture was stirred at room temperature until the completion of reaction (3 h) and monitored by TLC. The reaction mixture was then quenched with saturated aqueous $NaHCO_3$ (50 mL) and stirred for 5 min. The aqueous layer was extracted with CH_2Cl_2 (40 mL, 3 times) and the combined organic extracts were washed with brine (150 mL). After drying over sodium sulfate, filtration, washing with CH_2Cl_2 and evaporation of the solvent, the residue was purified by FC (AcOEt/MeOH 9:1) to give 252 mg (66 %) of **JJ08** (MW: 381.464) as white powder.

^{1}H NMR (400 MHz, $CDCl_3$) δH : 8.73 (br. s, 1H, HC(2vi), pyr)), 8.56 (m, 1H, HC(6iv), pyr)), 7.77 (d, 1H, 3J (HC(4''')-HC(5''')) = 6.7 Hz, HC(5'''), fur)), 7.61 (d, 1H, 3J (HC(2)-HC(3)) = 15.7 Hz, H-C(3), acryl), 7.47 (m, 1H, HC(4iv), pyr)), 7.34-7.26 (m, 1H, HC(5iv), pyr)), 6.92 (m, 1H, HC(3'''), fur), 6.53 (d, 1H, 3J(HC(2)-HC(3))) = 15.7 Hz, HC(2), acryl)), 6.46 (m, 1H, HC(4'''), fur)), 6.33 (br. s, 1H, HN-C(1'), aminobut), 4.50 (m, 2H, HC(2"a) & HC(6"a), piper), 3.39 (m, 2H, 3J (HC(1')-HC(2')) = 6.0 Hz, H2C(1'), aminobut), 3.16-2.65 (m, 2H, HC(2"e) & HC(6"e), piper), 1.18-1.72 (br. m), 1.62-1.49 (br. m), 1.44-1.13 (br. m).

^{13}C NMR (400 MHz, $CDCl_3$) δC : 165.2 (C=O), 159.2 (C=O), 150.2 & 149.1 (C(2iv) & C(6iv), pyr), 148.1 (C(2'''), fur), 143.5 (C(5'''), fur), 137.0 (C(3), acryl), 134.4 & 130.8 & 123.7 (pyr), 123.1 (C(2), acryl), 115.6 (C(3'''), fur), 111.0 (C(4'''), fur), 46.8 (C(2") & C(6"), piper), 39.7(C(1'), but), 36.1, 36.0, 32.4, 29.8, 23.9.

IR (KBr) ν: 3290, 3080, 2932, 2856, 1666, 1618, 1567, 1488, 1440, 1230, 981, 758 cm^{-1}.

HRMS (ESI) m/z [382 $(M+1)^+$]: 382.472.

^{13}C-NMR, infra-red (IR) and ^{1}H-NMR spectra of JJ08:

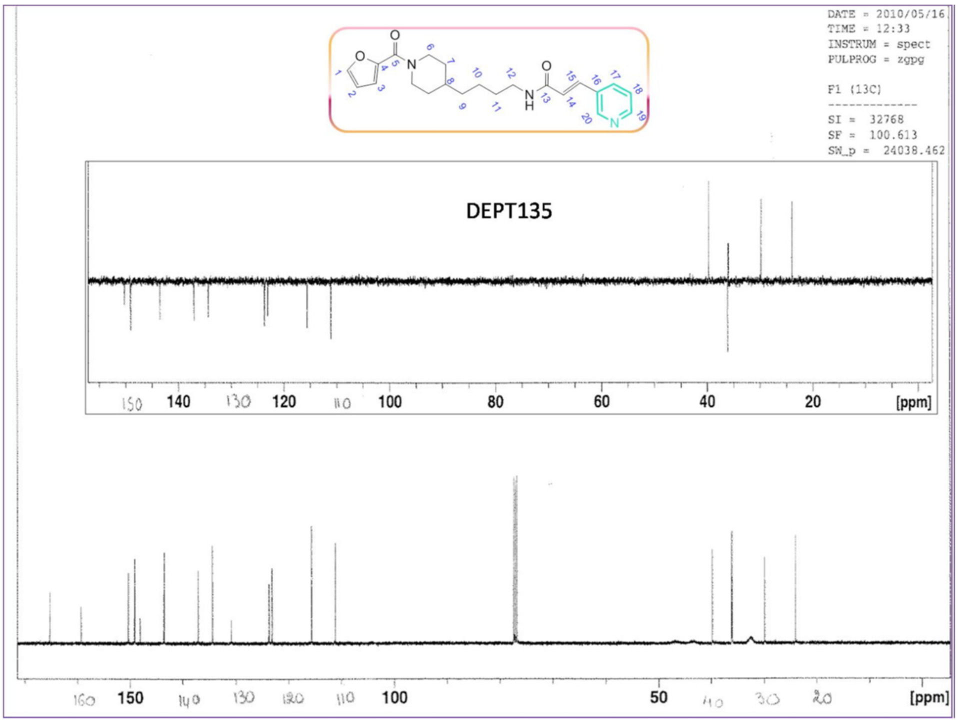

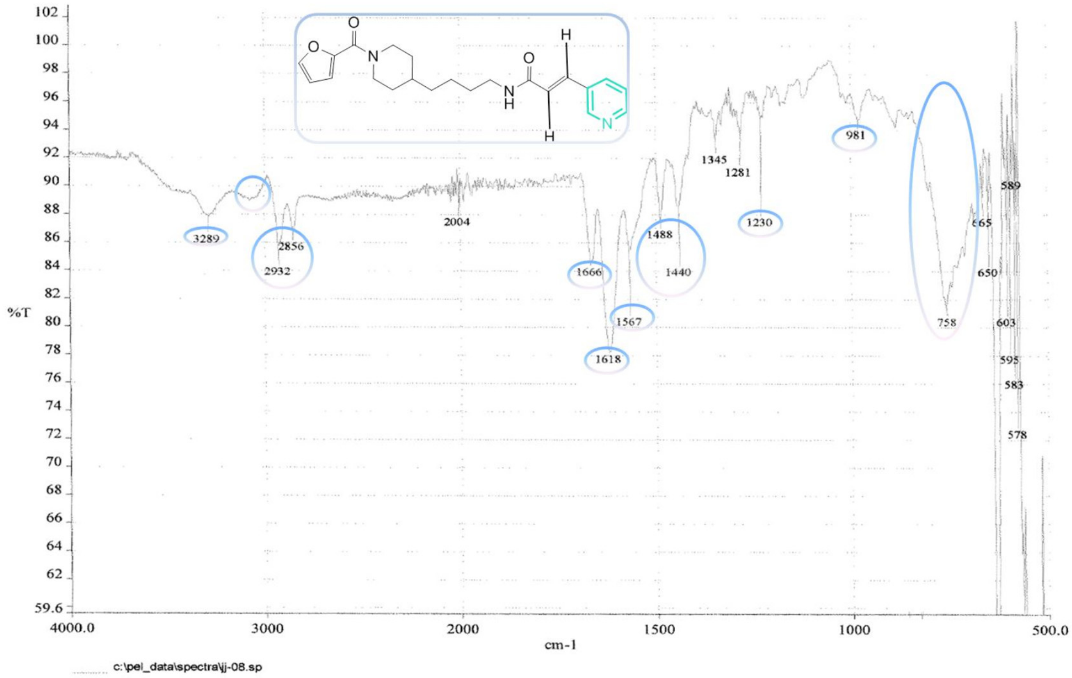

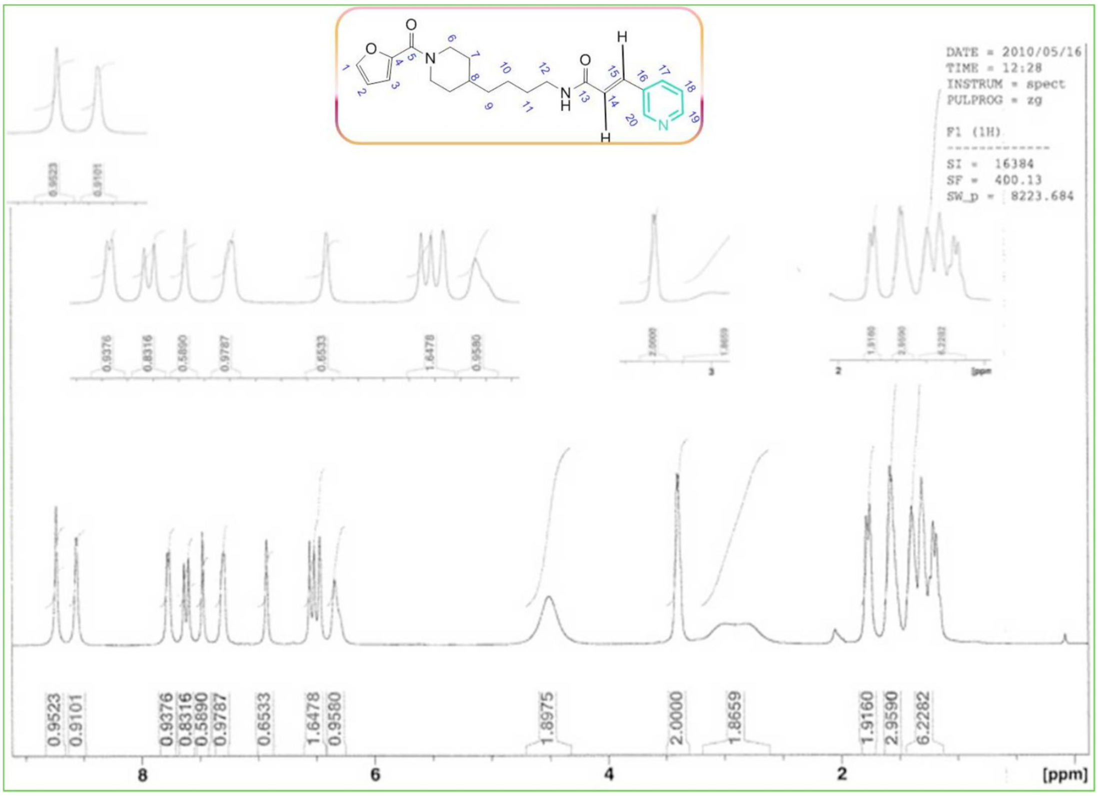

Part B: Syntheses of **FEI191** and **FEI199.**

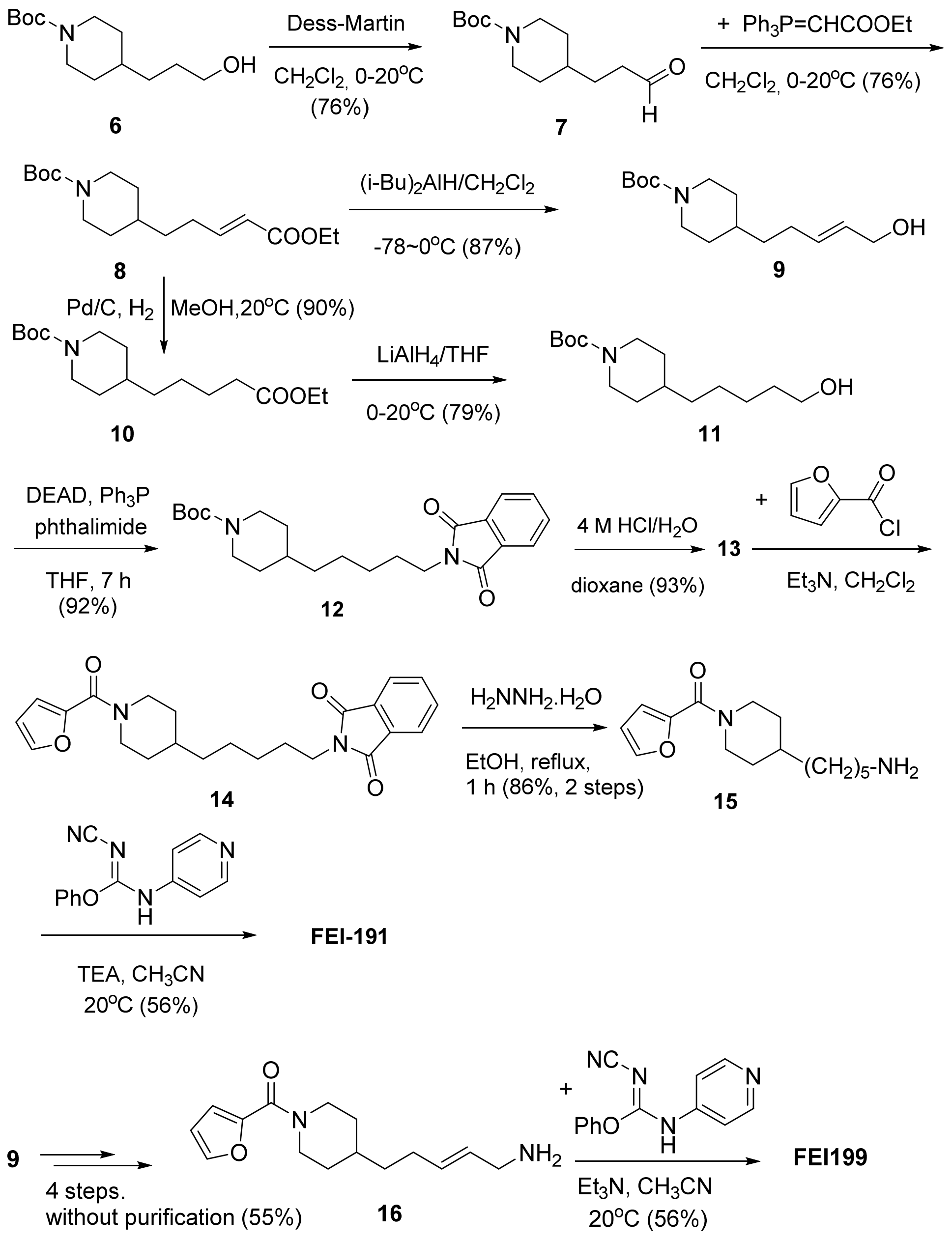

tert-Butyl 4-(3-oxopropyl)piperidine-1-carboxylate (7).

A solution of *tert*-butyl 4-(3-hydroxypropyl)piperidine-1-carboxylate (**6**, MW: 243.34; 1.00 g, 4.11 mmol, Ambeed, Inc. MDL number MFCD02677712) in CH_2Cl_2 (20 mL) was cooled to 0°C. 1,1,1-triacetoxy-1,1-dihydro-1,2-benziodoxol-3(1*H*)-one (Dess-Martin reagent, MDL nb. MFCD00130127, Sigma-Aldrich; 2.27g, 5.34 mmol) was added portionwise and the mixture was stirred at 20°C for 4 h. The reaction mixture was filtered, and the filtrate washed first with a saturated aqueous solution of $NaHCO_3$ (20 mL), then with brine (20 mL). After drying ($MgSO_4$) the solvent was evaporated *in vacuo*. The residue was purified by FC (PE/EtOAc 8:1) to give 0.85 g (86%) of **7** (MW: 241.331, CAS NO:165528-85-8), colorless oil that was used directly in the next step.

tert-Butyl (_E_)-4-(5-ethoxy-5-oxopent-3-en-1-yl)piperidine-1-carboxylate (8).

tert-Butyl 4-(3-oxoethyl)piperidine-1-carboxylate (**7**, 0.85 g, 3.5 mmol) was dissolved in CH_2Cl_2 (15 mL); (carbethoxymethylene)triphenylphosphorane (MW 348.37; 2.44 g, 7.0 mmol) was added in small

portions under vigorous stirring at 0ºC. The mixture was stirred at 20ºC for 3 h, then concentrated *in vacuo*. The residue was purified by FC (PE/EtOAc 5:1) to give 0.83 g (76%) of **8** (MW: 311.397) as colorless oil.

^{1}H NMR (400 MHz, $CDCl_3$): δH = 6.97 (dt, 1H, J = 15.6 Hz, 6.8 Hz, HC(3"), acryl), 5.84 (dt, 1H, J = 15.6 Hz, 1.4 Hz, HC(4"), acryl), 4.21 (q, 2H, J = 7.1 Hz, H_2C of ethoxy), 4.16-4.03 (m, 2H), 2.68 (m, 2H, J = 10.9 Hz, HC(2'a) & HC(6'a), piper), 2.25 (br. q, 2H, J = 6.8 Hz, H2C(2")), 1.71-1.63 (m, 2H), 1.47 (s, 9H, t-but), 1.46-1.37 (m, 5H), 1.31 (t, 3H, J = 7.1 Hz, CH_3 of ethoxy), 1.11 (qd, 2H, J = 10.7 Hz, 4.0 Hz).

***tert*-Butyl (*E*)-4-(5-hydroxypent-3-en-1-yl)piperidine-1-carboxylate (9).**

1M DIBAL in CH_2Cl_2 (10 mL) added slowly to ester **8** (1.45 g, 4.68 mmol) in CH_2Cl_2 (10 mL) under N_2 atmosphere at -78ºC over 20 min. The mixture was stirred at -78ºC for 40 min (monitored by TLC), and methanol (2.0 mL) was added dropwise to quench the excess of DIBAL. The cold solution was further stirred for 10 min before pouring it into a saturated sodium, potassium tartrate salt solution (30 mL). The mixture was then vigorously stirred at 20ºC for 3 h until it turned clear. The organic phase was collected and dried ($MgSO_4$), concentrated *in vacuo* to give 1.10 g (87%) of **9** (MW: 269.361) as colorless oil, which was used in the next step without purification.

^{1}H NMR (400 MHz, $CDCl_3$): δH = 5.76-5.62 (m, 2H, HC(3") & HC(4")), 4.12 (m, 2H, J = 5.3 Hz, H2C(5")), 4.09-3.99 (m, 1H, HO), 2.69 (m, 2H, J = 13.0 Hz), 2.10 (m, 2H, J = 14.1 Hz, 6.6 Hz), 1.75-1.62 (m, 2H), 1.47 (s, 9H, *t*-but), 1.45-1.30 (m, 5H), 1.31-1.25 (m, 2H), 1.10 (qd, 2H, J = 11.8 Hz, 3.9 Hz).

***tert*-Butyl 4-(5-ethoxy-5-oxopentyl)piperidine-1-carboxylate (10).**

Ene-ester **8** (0.6 g, 1.92 mmol) was dissolved in methanol (5 mL), then Pd/C (21.3 mg) was added under N_2 atmosphere. The reaction mixture was degassed and H_2 was introduced. The mixture was stirred under H_2 atmosphere at 20ºC overnight. The suspension was filtered on a Celite bed and the cake was washed with MeOH (15 mL). After evaporation of the solvent *in vacuo*, 0.54 g (89.6%) of **10** (MW: 313.411) was obtained as yellow oil, which was used directly in the next step without purification.

^{1}H NMR (400 MHz, $CDCl_3$): δH = 4.15 (q, 2H, J = 7.0 Hz, H_2C of ethoxy), 4.12-4.02 (m, 2H), 2.68 (br. t, 2H, J = 12.5 Hz), 2.32 (br. t, 2H, J = 7.6 Hz), 1.71-1.55 (m, 6H), 1.47 (s, 9H, *t*-but), 1.42-1.32 (m, 3H), 1.27 (t, 3H, J = 7.0 Hz, CH_3 of ethoxy), 1.08 (m, 2H, J = 11.9 Hz, 4.1 Hz).

***tert*-Butyl 4-(5-hydroxypentyl)piperidine-1-carboxylate (11).**

To a solution of **10** (0.54 g, 1.72 mmol) in THF (5 mL) was added lithium aluminum hydride (0.2 g, 5.16 mmol) in small portions at 0ºC. Then the mixture was stirred at 20ºC overnight (monitored by TLC). After the end of the reaction, the mixture was cooled to 0°C, and 30% aq. KOH (5 mL) was added slowly under vigorous stirring. After further stirring at 20°C for 1 h, the suspension was filtered on a Celite bed and the cake was washed with THF (15 mL). Evaporation of the solvent *in vacuo*, the residue was purified by FC (PE/EtOAc 3:1) to give 0.37 g (79 %) of **11** (MW: 271.377) as yellow oil, used directly in the next step.

^{1}H NMR (400 MHz, $CDCl_3$): δH = 4.18-3.95 (m, 2H), 2.81-2.59 (m, 4H), 1.70-1.62 (m, 2H), 1.47 (s, 9H), 1.38-1.29 (m, 6H), 1.30-1.21 (m, 3H), 1.08 (m, 2H, J = 12 Hz, 4.0 Hz).

***tert*-Butyl 4-(5-(1,3-dioxoisoindolin-2-yl)pentyl)piperidine-1-carboxylate (12).**

Under N_2, phthalimide (0.226 g, 1.54 mmol) and Ph_3P (0.404 g, 1.54 mmol) were dissolved in dry THF (5 mL). This solution was added to a solution of **11** (0.37 g, 1.36 mmol) in dry THF (5 mL). The reaction

mixture was stirred at 20°C for 10 min then cooled to 0°C. Diethyl azodicarboxylate (0.27 g, 1.54 mmol) was added dropwise at 0°C. Then the cooling bath was removed and the reaction mixture was stirred at 20°C for 7 h (monitored by TLC). The solvent was evaporated *in vacuo*, and the residue was purified by FC (Acetone/PE 1:10) to give 0.501 g (92%) of **12** (MW: 400.491), yellowish solid, used directly in the next step.

Hydrochloride of 4-(5-(1,3-dioxoisoindolin-2-yl)butyl)piperidine (13).

Solution of **12** (0.5 g, 1.24 mmol) in 4M HCl/dioxane solution (1.0 mL was stirred at 20°C for 30 min (monitored by TLC). After evaporation of the solvent *in vacuo*, 0.371 g (93%) of compound **13** (MW: 321.819) was obtained as white solid that was used directly in the next step without purification.

(4-(5-(1,3-dioxoisoindolin-2-yl)pentyl)piperidin-1-yl)(furan-2-yl)methanone (14).

A solution of **13** (371 mg, 1.15 mmol) in CH_2Cl_2 (20 mL) was cooled to 0°C, added triethyl amine (400 mg, 4 mmol), the mixture was stirred for 30 min, then 2-furoylchloride (160 mg, 1.2 mmol) in CH_2Cl_2 (10 mL) was added dropwise to the reaction mixture at 0°C. The mixture was then stirred at 20°C for 3 h. Saturated aqueous solution of $NaHCO_3$ (40 mL) was added and the mixture stirred vigorously for 5 min. The aqueous layer was extracted with CH_2Cl_2 (30 mL, 3 times) and the combined organic extracts were washed with brine (100 mL). After drying ($MgSO_4$) and evaporation of the solvent *in vacuo*, crude **14** (MW: 394.449) was obtained which was directly used in the next step.

(4-(5-Aminopentyl)piperidin-1-yl)(furan-2-yl)methanone (15).

Under N_2, crude **14** obtained above and hydrazine hydrate (0.15 mL, 2.9 mmol) were dissolved in dry ethanol (7 mL). The mixture was stirred at 20°C for 10 min and then heated under reflux (85°C) for 2 h. During the reaction a white precipitate formed as by-product. After cooling down to 20°C, the white precipitate was filtered off and washed with additional ethanol (8 mL). Solvent evaporation *in vacuo* gave a slurry that was dissolved in CH_2Cl_2 (10 mL) and a saturated aqueous solution of K_2CO_3 (10 mL) was added giving a clear suspension. The aqueous layer was extracted with CH_2Cl_2 (5 mL, 3 times) and the combined organic extracts were washed with brine (10 mL). After drying ($MgSO_4$), the solvent was evaporated *in vacuo*, and the residue was purified by FC (MeOH/EtOAc/TEA 80:18:2) to give 0.261 g (86%, two steps, based on **13**) of **15** (MW: 264.347) as yellow oil.

^{1}H NMR (400 MHz, $CDCl_3$): δH = 7.49 (br. d, 1H, J = 1.7 Hz, HC(5iv)), 6.95 (br. d, 1H, J = 3.4 Hz, HC(3iv)), 6.48 (dd, 1H, J = 3.4 Hz, 1.7 Hz, HC(4iv), fur), 4.76-4.34 (m, 2H), 3.22-2.77 (m, 2H), 2.72 (br. t, 2H, J = 7.0 Hz, HC(5'''), CH_2-NH_2), 1.84-1.75 (m, 2H), 1.60-1.43 (m, 3H), 1.42-1.35 (m, 4H), 1.35-1.17 (m, 4H).

Phenyl (Z)-N'-cyano-N-(pyridin-4-yl)carbamimidate.

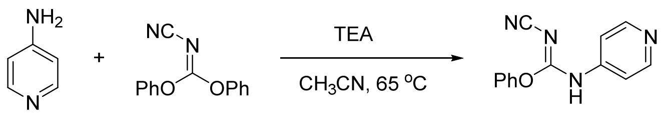

This compound was prepared according to a literature procedure [Tagmose, T. M.; Schou, S. C.; Morgensen, J. P.; Nielsen, F. E.; Arkhammar, P. O. G.; Wahl, P.; Hansen, B. S.; Worsaae, A.; Boonen, A. C. M.; Antoine, M. H. *J. Med. Chem.* **2004**, *47*, 3202-3211].

A solution of pyridin-4-amine (0.47g, 5.0 mmol), diphenyl *N*-cyanocarbonimidate (1.19g, 5.0 mmol) in acetonitrile (10 mL) was stirred at 65°C for 5 h (monitored by TLC). The reaction mixture was concentrated under reduced pressure and the residue was purified by FC (EtOAc) to give title compound as white solid (0.65 g, 55%).

^{1}H NMR (400 MHz, $CDCl_3$): δH= 8.41 (d, 2H, J = 4.0 Hz), 7.51 (dd, 2H, J = 5.0 Hz, 1.4 Hz), 7.47 (t, 2H, J = 8.0 Hz), 7.34 (t, 1H, J = 7.4 Hz), 7.21 (d, 2H, J = 7.8 Hz).

(*E*)-2-Cyano-1-(5-(1-(furan-2-carbonyl)piperidin-4-yl)pentyl)-3-(pyridin-4-yl)guanidine (FEI-191).

A solution of amine **15** (39.6 mg, 0.15 mmol), phenyl (*Z*)-*N'*-cyano-*N*-(pyridin-4-yl)carbamimidate (35.7 mg, 0.15 mmol) and triethylamine (20.8 µL, 0.15 mmol) in acetonitrile (2 mL) was stirred at 20ºC for 36 h (monitored by TLC). After the end of the reaction, the volatiles were evaporated under reduced pressure. The residue was purified by FC (EtOAc/MeOH 5:1) to give 34.5 mg (56.3%) of **FEI-191** (MW: 408.48), as a white foam.

^{1}H NMR (400 MHz, $CDCl_3$): δH = 8.43-8.42 (m, 2H), 7.46-7.45 (m, 1H), 7.26-7.23 (m, 2H), 6.87 (d, 1H, J = 3.4 Hz), 6.46 (q, 1H, J = 1.6 Hz), 6.13 (t, 1H, J = 1.6 Hz), 4.46 (brs, 2H), 3.39 (q, 2H, J = 6.4 Hz), 3.02-2.77 (m, 2H), 1.77-1.73 (m, 2H), 1.60-1.50 (m, 3H), 1.34-1.30 (m, 4H), 1.26-1.23 (m, 2H), 1.15 (qd, 2H, J = 12.4 Hz, 4.0 Hz); ^{13}C NMR (100 MHz, $CDCl_3$): δC = 159.3, 157.5, 150.5, 147.7, 145.2, 143.8, 117.0, 115.7, 115.6, 111.3, 47.2, 43.4, 42.6, 36.2, 36.0, 32.8, 32.3, 29.3, 26.9, 26.2; HRMS (ESI) for $C_{22}H_{28}N_6O_2$ [M+H]+ calcd: 409.2352, found: 409.2348.

^{1}H- and ^{13}C-NMR spectra of FEI-191:

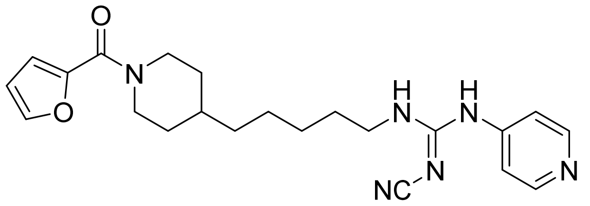

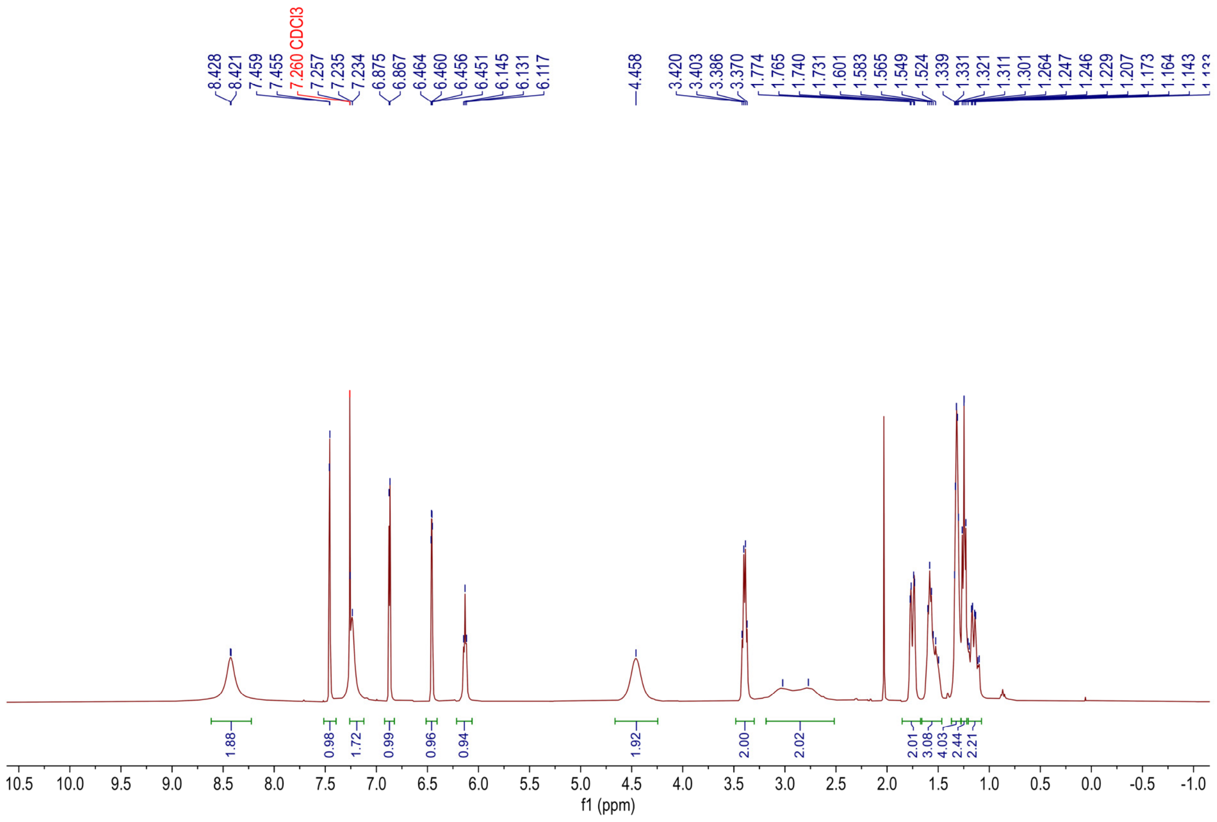

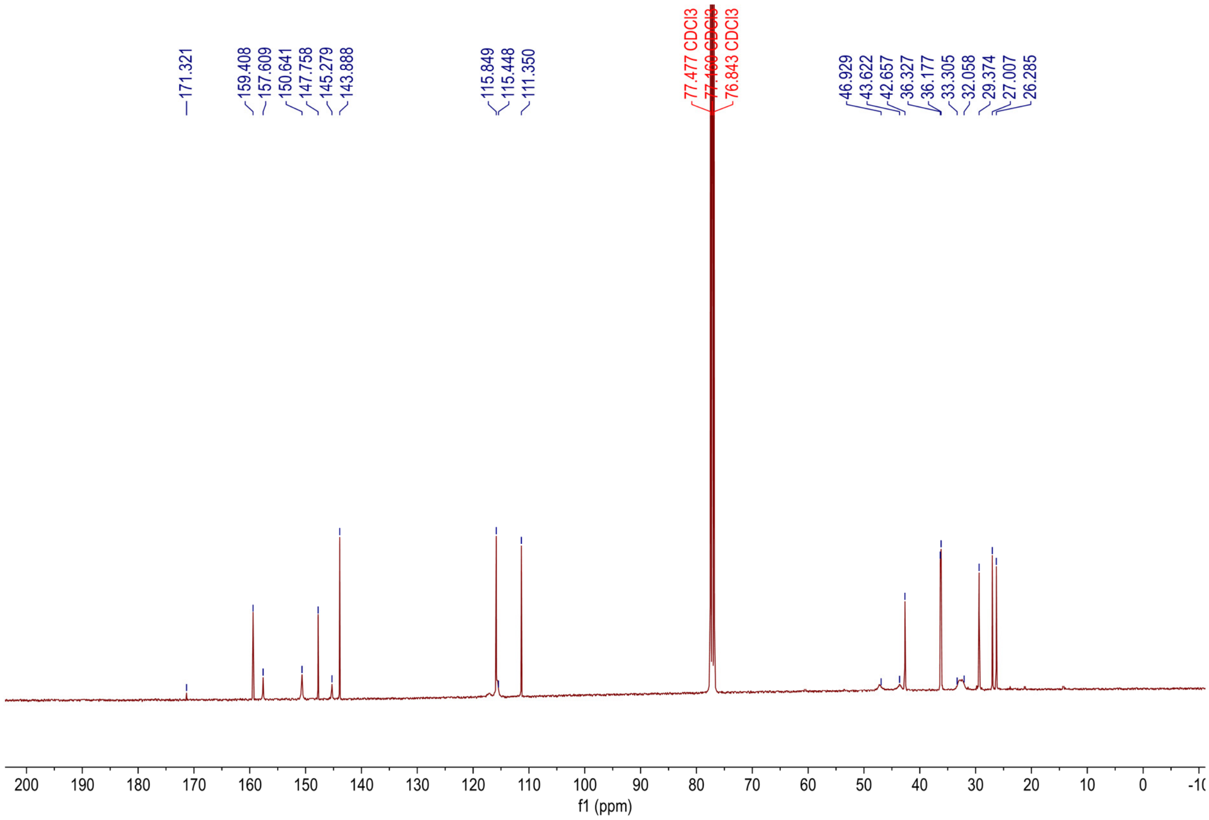

(*E*)-(4-(5-Aminopent-3-en-1yl)piperidin-1-yl)(furan-2-yl)methanone (16).

Crude **16** (MW: 262.333) was prepared from alcohol **9** (0.53g, 1.97 mmol) following the same procedure as that used to prepare **15** from **11**. Yield: 245 mg (55%, 4 steps). This crude product was used directly in the next step.

(*E*)-2-Cyano-1-((*E*)-5-(1-(furan-2-carbonyl)piperidin-4-yl)pent-2-en-1-yl)-3-(pyridin-4-yl)guanidine (FEI-199).

FEI-199 (MW: 406.466) was obtained from amine **16** (52.4 mg, 0.2 mmol) and phenyl (*Z*)-*N*'-cyano-*N*-(pyridin-4-yl)carbamimidate (47.6 mg, 0.2 mmol) according to the same procedure as that used to prepare **FEI-191**. Yield: 45.7 mg (56.3%), white foam.

^{1}H NMR (400 MHz, $CDCl_3$): δH = 8.47 (br. s, 2H), 7.46-7.45 (m, 1H), 7.24 (m, 2H), 6.90-6.88 (m, 1H), 6.46 (dd, 1H, J = 3.2 Hz, 1.6 Hz), 6.15 (br. s, 1H), 5.74 (dt, 1H, J = 15.6 Hz, 6.8 Hz), 5.50 (dt, 1H, J = 15.6 Hz, 6.0 Hz), 4.48 (br. s, 2H), 3.96 (t, 2H, J = 6.0 Hz), 2.98-2.79 (m, 2H), 2.13-2.07 (m, 2H), 1.78-1.74 (m, 2H), 1.61-1.52 (m, 2H), 1.38-1.33 (m, 3H), 1.19 (qd, 2H, J = 12.4 Hz, 4.0 Hz); ^{13}C NMR (100 MHz, $CDCl_3$): δC = 171.8, 159.4, 157.8, 150.9, 147.9, 144.8, 143.8, 135.5, 124.6, 115.9, 115.6, 111.3, 47.1, 44.5, 43.6, 36.8, 35.8, 33.0, 33.3, 29.4; HRMS (ESI) for $C_{22}H_{26}N_6O_2$ $[M+H]^+$ calcd: 407.2195, found: 407.2195.

^{1}H-NMR spectrum of FEI199

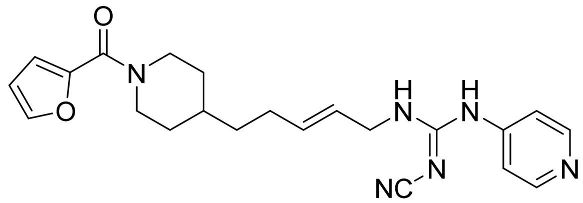
O
N
H
N
H
N
N
NC
N
O

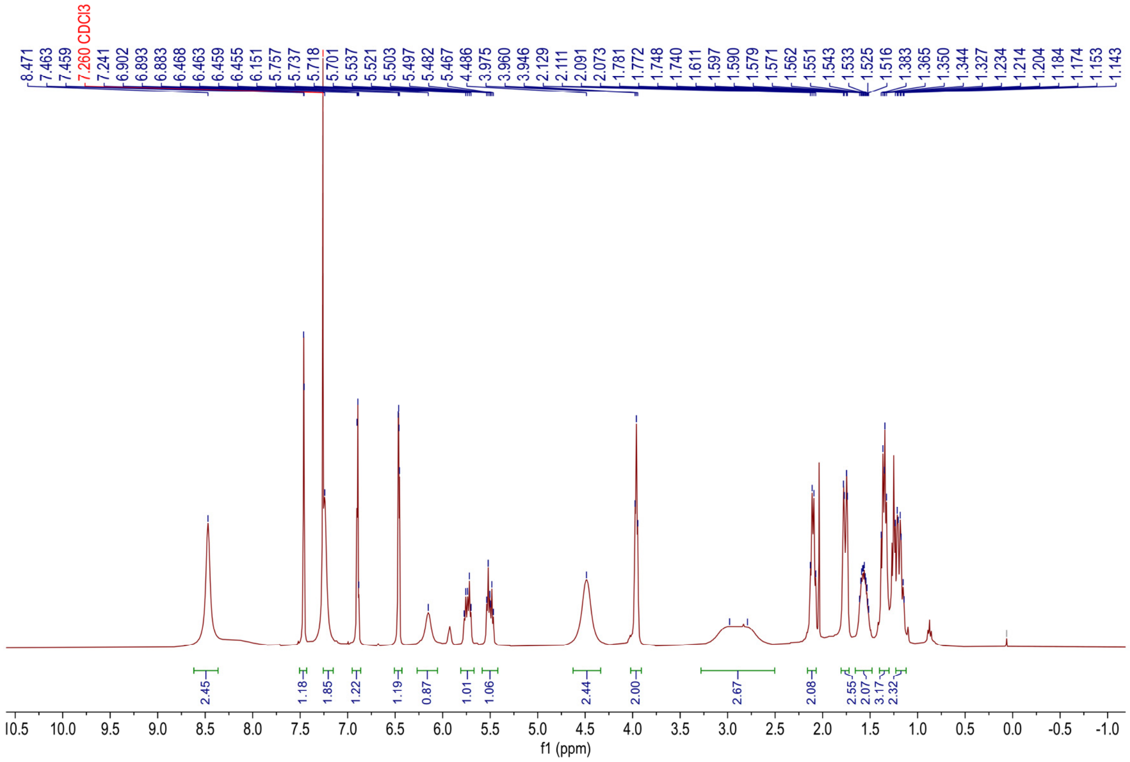
8.471
7.463
7.459
7.260 CDCl3
7.241
6.902
6.893
6.883
6.468
6.463
6.459
6.455
6.151
5.757
5.737
5.718
5.701
5.537
5.521
5.503
5.497
5.482
5.467
4.486
3.975
3.960
3.946
2.129
2.111
2.091
2.073
1.781
1.772
1.748
1.740
1.611
1.597
1.590
1.579
1.571
1.562
1.551
1.543
1.533
1.525
1.516
1.383
1.365
1.350
1.344
1.327
1.234
1.214
1.204
1.184
1.174
1.153
1.143
2.45
1.18
1.85
1.22
1.19
0.87
1.01
1.06
2.44
2.00
2.67
2.08
2.55
2.07
3.17
2.32
f1 (ppm)

Geometric means of plasma concentrations of the NAMPT inhibitors

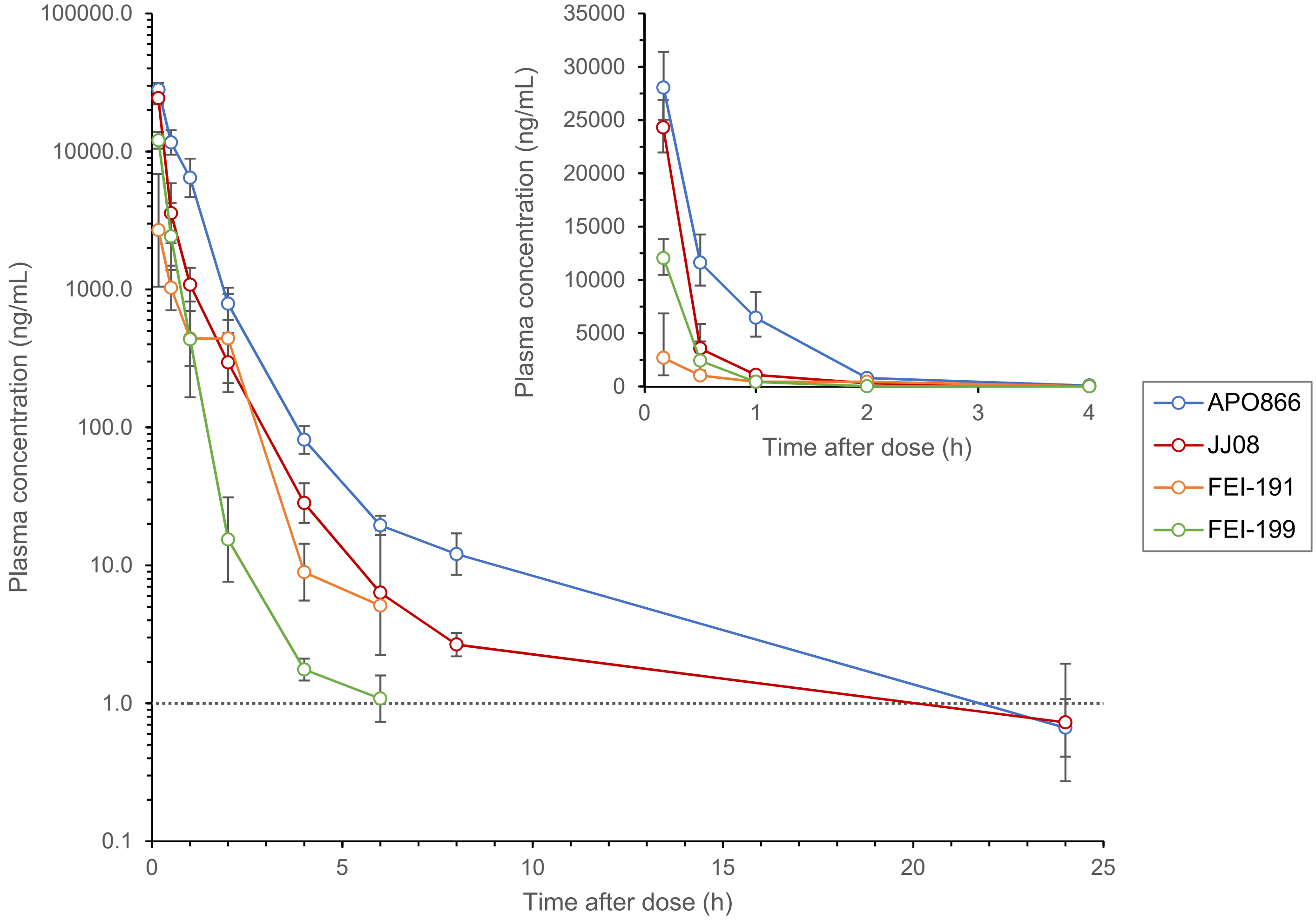

Figure S1: Geometric means of plasma concentrations of NAMPT inhibitors after intraperitoneal administration of drugs at 20 mg/kg (i.e. 0.4 mg per mouse for an average weight of 20 g per mouse). The horizontal dotted line represents the lower limit of quantification.

Compounds concentrations used for the PK analysis

Table S1: Measured concentrations of JJ08, APO866, FEI-191 and FEI-199.

Time after dose (h)	JJ08			APO866			FEI-191			FEI-199		
	Measured concentrations (ng/mL)	Mean concentration (ng/mL)	RSD (%)	Measured concentrations (ng/mL)	Mean concentration (ng/mL)	RSD (%)	Measured concentrations (ng/mL)	Mean concentration (ng/mL)	RSD (%)	Measured concentrations (ng/mL)	Mean concentration (ng/mL)	RSD (%)
	27571.2			28176.2			7931.8			10483.1		
0.17	22866.6	24310.5	11%	31307.2	28027.9	11%	1611.9	2682.8	137%	12005.1	12030.7	14%
	22789.0			24960.2			1510.3			13836.4		
	6124.2			14703.5			1546.9			2248.9		
0.50	2133.1	3630.5	55%	10108.0	11620.6	22%	932.3	1025.4	41%	4366.9	2418.3	62%
	3663.1			10558.4			747.6			1440.2		
	1295.9			4537.1			719.0			821.6		
1.00	766.6	1085.7	28%	6924.9	6440.9	31%	413.8	441.0	50%	143.2	434.3	83%
	1288.2			8504.4			288.4			696.6		
	533.1			615.4			767.2			9.1		
2.00	270.9	296.0	62%	751.2	785.9	28%	189.4	440.9	67%	34.3	15.4	90%
	179.6			1049.9			589.7			11.6		
	25.1			63.2			9.2			1.5		
4.00	42.8	28.3	41%	84.9	81.2	23%	5.5	8.9	49%	1.7	1.8	19%
	21.1			100.0			14.1			2.1		
	23.8			23.5						1.7		
6.00	2.7	6.3	187%	17.7	19.5	17%	5.1	5.1	1%	0.9	1.1	44%
	4.0			17.8			5.2			0.8		
	2.3			11.6								
8.00	3.2	2.7	17%	8.7	12.1	36%						
	2.7			17.4								
	0.6			0.6								
24.00	0.3	0.7	154%	1.1	0.7	55%						
	2.4			0.5								

RSD: relative standard deviation.
